# Characterizing Permethrin and Etofenprox Resistance in Two Common Laboratory Strains of *Anopheles gambiae* (Diptera: Culicidae)

**DOI:** 10.3390/insects9040146

**Published:** 2018-10-22

**Authors:** Aaron D. Gross, Jeffrey R. Bloomquist

**Affiliations:** Neurotoxicology Laboratory, Department of Entomology and Nematology, Emerging Pathogens Institute, University of Florida, Gainesville, FL 32611, USA; jbquist@epi.ufl.edu

**Keywords:** knockdown resistance, *kdr*, pyrethroid, insecticide, pseudo-pyrethroid

## Abstract

*Anopheles gambiae* Giles (Diptera: Culicidae) is the most prolific malaria vector in sub-Saharan Africa, where widespread insecticide resistance has been reported. *An. gambiae* laboratory strains are commonly used to study the basic biology of this important mosquito vector, and also in new insecticide discovery programs, where insecticide-susceptible and -resistant strains are often used to screen new molecules for potency and cross-resistance, respectively. This study investigated the toxicity of permethrin, a Type-I pyrethroid insecticide, and etofenprox, a non-ester containing pyrethroid insecticide, against *An. gambiae* at three life stages. This characterization was performed with susceptible (G3; MRA-112) and resistant (A*kdr*; MRA-1280) *An. gambiae* strains; the A*kdr* strain is known to contain the L1014F mutation in the voltage-sensitive sodium channel. Surprisingly, etofenprox displays a lower level of resistance than permethrin against all stages of mosquitoes, except in a headless larval paralysis assay designed to minimize penetration factors. In first-instar *An. gambiae* larvae, permethrin had significant resistance, determined by the resistance ratio (RR_50_ = 5), but etofenprox was not significantly different (RR_50_ = 3.4) from the wild-type strain. Fourth-instar larvae displayed the highest level of resistance for permethrin (RR_50_ = 108) and etofenprox (RR_50_ = 35). Permethrin (PC_50_ = 2 ppb) and etofenprox (PC_50_ = 9 ppb) resulted in headless larval paralysis (5-h), but resistance, albeit lower, was still present for permethrin (RR_50_ = 5) and etofenprox (RR_50_ = 6.9). In adult female mosquitoes, permethrin displayed higher resistance (RR_50_ = 14) compared to etofenprox (RR_50_ = 4.3). The level of etofenprox resistance was different from that previously reported for a similar Akron *An. gambiae* laboratory strain (MRA-913). The chemical synergists piperonyl butoxide (PBO) and diethyl maleate (DEM) were able to synergize permethrin, but not etofenprox in the resistant strain (A*kdr*). In conclusion, multiple mechanisms are likely involved in pyrethroid resistance, but resistance profiles are dependent upon selection. Etofenprox is an effective insecticide against *An. gambiae* in the lab but will likely suffer from resistance in the field.

## 1. Introduction

The African malaria mosquito, *Anopheles gambiae* Giles (Diptera: Culicidae), is the most efficient vector of malaria in Sub-Saharan Africa [1], and insecticide treated bed nets (ITNs) and/or indoor residual spraying (IRS) are used to decrease its populations. Reliance on chemical insecticides has resulted in widespread insecticide resistance to at least two insecticide classes [2] and is a continuous factor impeding the success of malaria elimination efforts [1]. Approximately 80% of the countries endemic with *An. gambiae* report resistance to at least a single class of insecticide, and more than 60% of these countries reported resistance to two or more insecticide classes [1]. Protection of military personnel from arthropod-vectored diseases is often achieved with the use of insecticide-treated combat and work uniforms [3,4], and permethrin, a Type-I pyrethroid insecticide, has long been approved for this use. The U.S. Environmental Protection Agency (EPA) has recently approved the so-called pseudo-pyrethroid etofenprox, which is a non-ester containing pyrethroid, for use on military uniforms [5]. 

According to the Insecticide Resistance Action Committee’s (IRAC’s) mode of action classification system, pyrethrum and pyrethroid insecticides (Group 3) modify the insect voltage-sensitive sodium channel (VSSC) [6]. VSSCs are transmembrane proteins that function in the movement of sodium ions into the cell, resulting in membrane depolarization during an action potential. Disruption of the VSSC by pyrethroids alters channel function by delaying inactivation; this action has been extensively studied and recently reviewed [7,8,9]. Different classes of pyrethroid insecticides differently affect the VSSC causing depolarization of resting membrane potential, and/or repetitive nerve firing [9]. 

Arthropods have evolved mechanisms to decrease the toxicity of pyrethroid insecticides. Mechanisms of pyrethroid resistance include increased metabolism of the insecticide by cytochrome P450-monooxygenases, general esterases, glutathione *S*-transferases, and/or target site modification resulting in reduced sensitivity [2,10,11,12,13,14]. Target site modification is characterized as the genotypic modification to the amino acid sequence resulting in an altered phenotype. For pyrethroid insecticides, genotypic modification of the VSSC results in a knockdown resistant (kdr) phenotype. In *An. gambiae*, a leucine to phenylalanine replacement at amino acid position 1014 (L1014F) in the *para*-type sodium channel is commonly associated with pyrethroid resistance [15]. Additional target site mutations have been reported in the VSSC from resistant *An. gambiae* populations and other insects [16,17].

The goal of this study was to characterize the toxicity of permethrin and etofenprox and determine the level of resistance in the *An. gambiae* Akron-*kdr* (Akdr; MRA-1280) strain, which is known to carry the L1014F mutation (and perhaps other mechanisms) resulting in pyrethroid resistance [18]. This study is an extension of previous work [2] with an emphasis on documenting the resistance observed with etofenprox. The characterization of etofenprox is particularly important based on the recent approval of its use on military uniforms [5].

## 2. Materials and Methods

### 2.1. Insects

*An. gambiae* were reared from eggs obtained from pyrethroid susceptible (G3, MRA-112) and pyrethroid-resistant (Akron-*kdr* (A*kdr*), MRA-1280) colonies maintained by the Malaria Research and Reference Reagent Resource Center (MR4), part of the Biodefense and Emerging Infections (BEI) Research Resources Repository at the Center for Disease Control and Prevention (CDC), Atlanta, GA, USA. Research with *An. gambiae* at the University of Florida was approved by the Florida Department of Agriculture and Consumer Services (FDACS permit #10-33), where *An. gambiae* were contained under BSL3/ACL3 conditions at the Emerging Pathogens Institute. Fourth-instar larvae of the MRA-1280 strain were selected with 1 ppm permethrin for 24-h and survivors used to maintain the resistant colony (personal communication with Paul Howell, 9 September 2015). *An. gambiae* eggs from either colony were placed into unfiltered tap water and fed a 2% (*w*/*v*) Brewer’s yeast suspension (MP Biomedicals, LLC, Santa Ana, CA, USA) for the first 12–24-h. First-instar larval density was between 500–1000 eggs in 500 mL of tap water. Larvae were then split into several pans at a density of 100–200 larvae per 1.2 L of tap water. The remaining larval life stages were fed pulverized beta-fish food (Spectrum Brands Holdings, Inc., Madison, WI, USA). Pupae were collected and placed into waxed paper containers with a mesh lid, where freshly emerged adults were provided a 10% sucrose water (*w*/*v*) solution via a soaked cotton ball ad libitum. All life stages were maintained in Percival incubators at 28 ± 2 °C with a relative humidity greater than 70% on a light: dark (12:12 h) photoperiod.

### 2.2. Chemicals and Chemical Preparation

Technical grade permethrin was purchased from Chem Services Inc. (West Chester, PA, USA). Etofenprox (99%, Pestanal^®^ analytical standard), piperonyl butoxide (PBO; 99%, Pestanal^®^ analytical standard), and diethyl maleate (DEM; >96%) were purchased from Sigma-Aldrich (St. Louis, MO, USA). Ethanol (>99%) was purchased from Fisher Scientific (Hampton, NH, USA). All test compounds and synergists were initially diluted into >99% ethanol (Fisher Scientific) before being serially diluted to the desired concentration (all in ethanol).

### 2.3. Larval Toxicity Assays

First-instar mortality bioassays with intact larvae were performed as previously described with minor modifications in Reference [19]. Control mortality or paralysis of all larval assays at all life stages was 10% or less. If greater than 10% control mortality was observed, the results were not used for analysis. Ten first-instar larvae (12–24-h post-hatch) were added to each well of a 24-well plate with 995 μL of tap water (containing 0.04% yeast suspension), followed by the addition of the test compound (5 μL) dissolved in ethanol (final ethanol concentration was 0.5%). To ensure even mixing of the treatments, the Petri dishes were swirled in clockwise and counterclockwise motions, along with front-and-back and side-to-side motions ten times. Wells containing ethanol only (0.5%) served as the negative control. Larval moribundity was determined 24-h post-treatment. Larvae that showed no movement after manual disturbance of the water by a pipette tip were scored as “dead”. 

Fourth-instar (intact) *An. gambiae* larval bioassays were performed in a similar fashion as the first-instar bioassays, but used a 35-mm Petri dish containing 4.75 mL of tap water prior to the addition of 25 μL of the test compound dissolved in ethanol. The final concentration of ethanol in all treatments and the negative control was 0.5%. Contents of the wells were mixed as previously described. Larval mortality was determined 24-h after the addition of test compounds or solvent control. Larvae that did not move when manually disturbed were recorded as dead. 

The paralytic effect of the test compounds was examined using a previously described headless larval assay [20,21] that was adapted for *An. gambiae.* Briefly, the heads of fourth-instar larvae were removed using forceps before being transferred to a 35-mm Petri dish containing 4.75 mL of mosquito larval physiological saline, as in References [20,22]. Test compounds, dissolved in ethanol (25 μL), were added to the Petri dish and mixed as previously described. Five-hours post-treatment, the paralytic effect of the test compounds and control was measured. Paralysis was scored if there was no movement or slight twitching after larvae were probed with an insect pin. 

### 2.4. Adult Topical Biological Assays

Non-blood fed adult female *An. gambiae* mosquitoes (1–5 days post-emergent) were anesthetized on ice, and treatments were applied (volume of 0.2 μL) to the mosquito’s thorax using a Hamilton ten-microliter gas-tight syringe equipped with a repeating dispenser (Hamilton Company, Reno, NV, USA). Ethanol (>99%) alone was used as the negative control. Synergism experiments were performed in a similar manner, where mosquitoes were dosed with piperonyl butoxide (PBO) or diethyl maleate (DEM) at 100 ng/mosquito; mosquito mortality at these synergist doses was less than 20%. Mosquitoes that survived the synergistic treatment were again anesthetized on ice before being topically treated with the pyrethroid insecticide or solvent control four-hours after the synergist treatment. The 1-h knockdown and 24-h moribundity were then determined. Knockdown (KD) was defined as mosquitoes that have erratic and uncontrollable flight or do not maintain proper posture when at rest; moribund mosquitoes were also included in this measurement at 1-h. Mosquito moribundity/mortality at 24-h was defined as no movement or only slight leg movements when the container was agitated.

### 2.5. Data Analysis

The half-maximal response for knockdown (KD_50_), lethality (LD_50_ or LC_50_), and paralysis (PC_50_) was calculated using the PROC PROBIT procedure in SAS 9.4 (SAS Institute, Carey, NC, USA). A minimum of five concentrations were tested per compound to generate a KD_50_, LD_50_, LC_50_, or PC_50_ and a minimum of three replicates were performed. A replicate was defined as treated mosquitoes obtained from different rearing cohorts. Statistical differences in knockdown, toxicity, or paralysis was performed by using individual determinants (LD_50_, LC_50_, or PC_50_) using Graphpad Prism 7.03 (La Jolla, CA, USA). First, data underwent logarithmic transformation then were examined to ensure that they fit a Gaussian distribution (Shapiro-Wilk normality test), before performing an unpaired t-test (α = 0.05). Resistance ratios (RR_50_) were calculated by dividing the KD_50_, LD_50_, LC_50_, or PC_50_ from the MRA-1280 strain by the corresponding values generated by the susceptible G3 (MRA-112) strain. Synergistic ratios (SR_50_) were calculated by dividing the LD_50_ obtained without synergist by the LD_50_ obtained with a synergist pre-treatment.

## 3. Results

### 3.1. Intact Larval Lethality Assays

The toxicity of permethrin and etofenprox was examined against susceptible (G3) and resistant (A*kdr*) first-instar *An. gambiae* larvae (Table 1). Permethrin was significantly more toxic to G3 first-instar larvae compared to A*kdr* (RR = 5) first-instar larvae (*p* = 0.0022). While etofenprox is numerically (RR = 3.4) more toxic to G3 than A*kdr* first-instar larvae, there was not a statistically significant difference (*p* = 0.104) between the two strains (Table 1). In G3 first-instar larvae, permethrin was significantly more toxic (3.7-fold) than etofenprox (*p* = 0.002), but permethrin was not significantly more toxic (2.5-fold) to A*kdr* first instar larvae compared to etofenprox (*p* = 0.346) (Table 1).

The toxicity of permethrin and etofenprox was examined against the two *An. gambiae* strains (G3 and A*kdr)* on intact fourth-instar larvae (Table 2). The resistance ratios for permethrin (RR = 108) and etofenprox (RR_50_ = 35) were much greater in fourth-instar larvae, when compared to first-instar larvae. Permethrin’s LC_50_ between the two strains (G3 and A*kdr*) was statistically different (*p* < 0.0001), with 100-fold lower LC_50_ observed in the G3 strain (Table 2). Similarly, the LC_50_ values for etofenprox against fourth-instar larvae were statistically different between the two strains (*p* < 0.0001), with higher toxicity observed in the G3 strain (Table 2). Unlike what was observed in first-instar *An. gambiae* larvae, the LC_50_s for permethrin and etofenprox were identical (*p* = 0.53) in G3 larvae. However, etofenprox resulted in a statistically significant lower LC_50_ value (*p* = 0.0014) in the A*kdr* strain compared to permethrin (Table 2).

### 3.2. Headless Fourth-Instar Larval Paralysis Assay

A headless fourth-instar larval assay was used to examine the paralytic activity of permethrin and etofenprox against decapitated *An. gambiae* that retain the ability to respond to probing (Table 3). Permethrin displayed 5-fold resistance between the two *An. gambiae* strains (G3 and A*kdr*), but it was not statistically significant (*p* = 0.099) (Table 3). A larger RR was observed for etofenprox (RR_50_ = 6.9); however, the PC_50_ values were not quite statistically significant (*p* = 0.0517) between the two strains (Table 3). Finally, the PC_50_ responses were not statistically significant for permethrin and etofenprox in the G3 strain (*p* = 0.1422) or A*kdr* strain (*p* = 0.5714) (Table 3).

### 3.3. Adult An. gambiae Knockdown (1-h) and Toxicity (24-h) Biological Assays

The knockdown and toxicity of permethrin and etofenprox were tested against adult female *An. gambiae* mosquitoes and are shown in Table 4 (G3) and Table 5 (A*kdr*). For both strains, the KD_50_ (1-h) dose for permethrin and etofenprox was not statistically different from the LD_50_ dose at 24-h. Permethrin resulted in a significantly lower KD_50_ (*p* < 0.0001) and LD_50_ (*p* = 0.0015) in the G3 strain compared to A*kdr* strain. Similarly, etofenprox had a significantly lower KD_50_ (*p* = 0.0099) and LD_50_ (*p* = 0.0161) for the G3 strain compared to the A*kdr* strain. 

Permethrin was tested with two chemical synergists (PBO and DEM) against the G3 strain (Table 4) and the A*kdr* strain (Table 5). In the G3 strain, a PBO pre-treatment with permethrin did not have a significant effect on the 1-h KD_50_ (*p* = 0.0992), but did affect the 24-h LD_50_ (*p* = 0.0030). DEM with permethrin did not have a significant effect in the G3 strain on the 1-h KD_50_ (*p* = 0.4164) or the 24-h LD_50_ (*p* = 0.170). PBO and DEM with permethrin had significant effects in the A*kdr* strain, where PBO significantly lowered the 1-h KD_50_ (*p* = 0.0327) and the 24-h LD_50_ (*p* = 0.0013). In the A*kdr* strain, DEM significantly lowered permethrin’s 1-h KD_50_ (*p* < 0.0001) and 24-h LD_50_ (*p* = 0.0019) in the A*kdr* strain. 

Etofenprox was also tested with the two synergists (PBO and DEM) against the G3 strain (Table 4) and A*kdr* strain (Table 5). Etofenprox with PBO (pre-treatment) in the G3 strain significantly lowered the 1-h KD_50_ (*p* = 0.0106) and the 24-h LD_50_ (*p* = 0.03). Similarly, DEM in the G3 strain significantly lowered the 1-h KD_50_ (*p* = 0.038) and the 24-h LD_50_ (*p* = 0.0411). However, PBO did not have a significant effect in the A*kdr* strain for the 1-h KD_50_ (*p* = 0.0673) or the 24-h LD_50_ (*p* = 0.0690). Etofenprox with a DEM (pre-treatment) in the A*kdr* strain also did not have a significant effect on the 1-h KD_50_ (*p* = 0.067) or the 24-h LD_50_ (*p* = 0.37). 

## 4. Discussion

The objective of this study was to examine the toxicity and the level of resistance of two pyrethroid insecticides against two laboratory strains of *An. gambiae* at three life stages (first-instar larvae, fourth-instar larvae, and adult female). Previous characterization of the *An. gambiae* WHO Akron strain (MRA-913) was performed using permethrin, deltamethrin, etofenprox, and DDT. Resistance was observed for all of these VSSC-acting insecticides except etofenprox, which had a RR_50_ of 1.4 [2]; these data were perplexing and needed further examination. It’s important to note the differences between the WHO Akron (MRA-913) and A*kdr* (MRA-1280) strains. These strains were isolated in the Akron District of Porto Novo, Benin (Africa). MRA-913 resistance to carbamate insecticides (phenotype) is a result of a genotype modification in the acetylcholinesterase enzyme (ACE-1 mutation) [2] and is selected in the laboratory with bendiocarb at the adult stage (personal communication with Paul Howell, 9 September 2015). Whereas, the MRA-1280 is selected with permethrin in the larval stage (previously stated in Materials and Methods). The selection in the laboratory is the only difference between these two *An. gambiae* strains; both strains display carbamate and pyrethroid resistance. 

The first-instar *An. gambiae* larval bioassay was developed to evaluate the toxicity of insecticides [19]. The benefit of this assay is that it has relatively high-throughput, eliminating the need to rear mosquitoes to older larvae or adults. Additionally, due to the small size of first-instar larvae a smaller water volume was used; therefore, less test compound was needed to perform the assay. This is an important advantage in an insecticide discovery program, where the amount of the test compounds may be limited. The caveat to this first-instar bioassay was that these small larvae were more susceptible to xenobiotics and may not provide an accurate prediction of mortality for later instars or adults. This effect was recently highlighted by comparing the toxicity of fluralaner to *Aedes aegypti* mosquito larvae [23]. Fluralaner’s toxicity to *Ae. aegypti* fourth-instar larvae was found to be 1.8 ppb [23]; however, a previous study reported greater than 90% mortality to first instar *Ae. Aegypti* larvae at 1.2 ppt [24], a greater than 1500-fold difference in toxicity between the two life stages. While we did not observe such a drastic difference between life stages for permethrin or etofenprox (Table 1 and Table 2), we did see differences between the larval life stages. For instance, in the G3 strain, permethrin was 31-fold more toxic to first instar larvae and etofenprox 8.4-fold more toxic to first instar larvae, when compared to fourth-instar (intact) larvae. Larger differences between the larval life stages were observed in the A*kdr* strain, where permethrin and etofenprox had a 667-fold and an 86-fold difference between first-instar and fourth-instar larvae, respectively. These differences in toxicity need to be taken into consideration when using the first-instar larval assay. 

The large difference in life stage susceptibility to permethrin and etofenprox indicates that physiological factors (size and weight) play a role; this was previously reported in *Culex quinquefasciatus,* where the larval instar correlated with susceptibility to permethrin toxicity [25]. One physiological factor that likely plays a role in the susceptibility to xenobiotics between life stages is the development of the cuticle. The importance of the cuticular barrier is demonstrated with the headless larval assay, which allows a direct diffusion pathway of test compounds; thereby facilitating penetration of the toxicant to exert its toxicodynamic effect without the need to cross the cuticular barrier. Curiously, the headless fourth-instar larvae had 5-h PC_50_ values (Table 3) similar to the 24-h LC_50_ values obtained with first-instar larvae (Table 1). These values were dramatically different from intact fourth-instar larvae (Table 2). It is likely that there is thickening of the cuticle between life stages, but cuticular composition may also differ between susceptible and resistant strains. In adult *An. gambiae*, changes in the expression of two P450 enzymes changed the cuticular hydrocarbon production on the cuticle of resistant adult mosquitoes [26]. As a result, there was a decrease in the penetration of pyrethroid insecticides. However, it is not yet clear what form of cuticular changes might occur in *An. gambiae* larvae, if any. 

Topical application of permethrin and etofenprox was performed in adult female *An. gambiae* mosquitoes. Permethrin’s toxicity aligned with previously reported topical data in the same species, with minor differences that were likely related to the health-status or rearing conditions of the colony [2,27]. When *An. gambiae* Akron mosquitoes are selected with permethrin, instead of bendiocarb, there appears to be slightly more resistance to pyrethroid insecticides [2], which is not surprising. To-date, only the L1014F mutation has been characterized in either the WHO-Akron (MRA-913) or A*kdr* (MRA-1280) strains [2,18]. However, other mutations have been reported in the VSSC of field-collected *An. gambiae* [28]. Intense selective pressure with insecticides on *An. gambiae* mosquitoes will ultimately result in the development of multiple types of insecticide resistance [11], including the potential for further mutations in the VSSC. 

Previously we reported an increase in the general esterase and cytochrome P450 *O*-deethylation activities of WHO Akron *An. gambiae* (MRA-913), compared to G3. While these biochemical assays have yet to be performed in the A*kdr* strain (MRA-1280), we did conduct toxicity assays using PBO (an inhibitor of P450 monooxygenases) and DEM (an inhibitor of glutathione *S*-transferases; GSTs). Esterase inhibitor (e.g., *S*,*S*,*S*-tributyl phosphorotrithioate) studies were not performed because esterase metabolism of etofenprox is unlikely, since it is a non-ester containing pyrethroid. Permethrin was synergized by PBO in the G3 strain and by PBO and DEM in the A*kdr* strain. These results indicated that metabolism of permethrin by cytochrome P450s and GSTs were likely mechanisms involved in resistance. In G3 mosquitoes, PBO and DEM were able to synergize etofenprox significantly, but the effect was not significant in the Akdr strain. These results suggested that there was an increase in phase I and II metabolism in the wild-type strain that was not present in the resistant (A*kdr*) strain. These results are baffling, since it would be expected that higher metabolic activities would be found in the resistant strain rather than the susceptible strain. Furthermore, PBO should synergize etofenprox toxicity, and this was previously reported in field-caught *An. funestus* mosquitoes [28]. It has been reported that the lipophilic nature of the chemical synergist PBO enhances penetration of deltamethrin allowing it to reach its target site before being metabolized [29]. However, the lack of PBO synergism with etofenprox in the A*kdr* strain makes this mechanism unlikely. Other factors that could affect etofenprox’s cuticular penetration, such as changes in cuticular components or transporters (ABCs), may be involved and have been reported in resistant insects [30]. Previously, the ZANDS *An. gambiae* strain, which possesses organochloride resistance via elevated GST activity, was tested against etofenprox. Etofenprox did not display any resistance (RR_50_ 0.89) in this mosquito strain, supporting the lack of DEM synergistic activity that we observed in the A*kdr* strain. The WHO-Akron (MRA-913) strain displayed resistance to DDT which was attributed to target site insensitivity, since there was not an increase in GST activity in biochemical assays [2]. Collectively, the known target site modification (L1014F) along with the studies performed with synergists and the headless larval assay indicate that multiple mechanisms (*kdr*, metabolism, and cuticle thickness) are likely involved in pyrethroid resistance of the A*kdr* strain; a similar conclusion made with the WHO Akron strain [2]. 

## 5. Conclusions

Laboratory reared mosquitoes are important for investigating the basic biology of mosquito populations that have a global impact, but may not be locally present. These strains are also important in insecticide discovery programs where cross-resistance can be identified early in the process. Laboratory selection is needed to help maintain resistance, but it is important to remember that this selection process can result in different resistance profiles. We conclude that multiple mechanisms of insecticide resistance are likely present in the A*kdr An. gambiae* strain, similar to what has been shown in the WHO Akron Strain [2]. The low levels of etofenprox resistance, and lack of resistance in MRA-913, was surprising, especially since previous reports have shown that other laboratory-maintained or field-caught mosquitoes display high levels of resistance to etofenprox [3,4,12,13,31,32]. While the recent approval of etofenprox for treatment of military uniforms may provide protection from *An. gambiae* in the laboratory, the ability of etofenprox to provide protection from *An. gambiae* mosquitoes, and the diseases they vector in the field is debatable.

## Figures and Tables

**Table 1:** First-instar toxicity of permethrin (PM) and etofenprox (EF).

	*An. gambiae* (G3)				*An. gambiae* (A*kdr*)				
	n	LC_50_^1^	Slope ± SE	χ^2^ (df)	n	LC_50_^1^	Slope ± SE	χ^2^ (df)	RR ^2^
PM	220	3 (2–4)	2.49 ± 0.54	76 (20)	220	15 (8–25)	1.75 ± 0.35	63 (20)	5
EF	340	11 (9–16)	2.04 ± 0.28	50 (32)	220	37 (18–88)	1.65 ± 0.48	84 (20)	3.4

^1^ LC_50_ values represent **ppb** of toxicant with the 95% CI shown in parentheses; ^2^ Resistance Ratio (RR_50_) was calculated by dividing the LC_50_ of the A*kdr* strain by that of the G3 strain.

**Table 2:** Fourth-instar (intact) toxicity of permethrin (PM) and etofenprox (EF).

	*An. gambiae* (G3)				*An. gambiae* (A*kdr*)				
	n	LC_50_^1^	Slope ± SE	χ^2^ (df)	n	LC_50_	Slope ± SE	χ^2^ (df)	RR ^2^
PM	450	0.093 (0.067–0.141)	1.40 ± 0.22	97 (42)	250	10 (8–14)	1.60 ± 0.21	30 (23)	108
EF	400	0.092 (0.077–0.109)	2.48 ± 0.25	53 (38)	420	3.2 (2.7–3.7)	1.95 ± 0.17	39 (40)	35

^1^ LC_50_ values represent **ppm** of toxicant with the 95% CI shown in parentheses; ^2^ Resistance Ratio (RR_50_) was calculated by dividing the LC_50_ of the A*kdr* strain by that of the G3 strain.

**Table 3:** Fourth-instar (headless) larvae paralytic effect of permethrin (PM) and etofenprox (EF).

		*An. gambiae* (G3)			*An. gambiae* (A*kdr*)				
	n	PC_50_^1^	Slope ± SE	χ^2^ (df)	n	PC_50_	Slope ± SE	χ^2^ (df)	RR ^2^
PM	140	2 (1–4)	0.67 ± 0.12	6 (12)	120	10 (3–27)	0.67 ± 0.12	11 (10)	5
EF	150	9 (5–18)	0.78 ± 0.12	15 (13)	130	62 (35–102)	1.28 ± 0.21	5 (11)	6.9

^1^ PC_50_ values represent **ppb** of toxicant with the 95% CI shown in parentheses; ^2^ Resistance Ratio (RR_50_) was calculated by dividing the PC_50_ of the A*kdr* strain by that of the G3 strain.

**Table 4:** Toxicity and synergism of permethrin (PM) and etofenprox (EF) *An. gambiae* (Strain G3).

	n	KD_50_, ng/mg (95% CI)	Slope (±SEM)	χ^2^ (df)	SR ^1^	LD_50_, ng/mg (95% CI)	Slope (±SEM)	χ^2^ (df)	SR ^1^
PM	286	0.13 (0.09–0.25)	2.15 (±0.48)	94 (24)	-	0.13 (0.08–0.29)	1.42 (±0.33)	76 (24)	-
+PBO	177	0.05 (0.03–0.18)	1.45 (±0.44)	41 (13)	2.6	0.02 (0.01–0.05)	1.32 (±0.35)	32 (14)	6.5
+DEM	169	0.10 (0.08–0.12)	2.91 (±0.38)	16 (14)	1.3	0.07 (0.04–0.10)	2.54 (±0.52)	29 (14)	1.9
EF	165	0.12 (0.005–0.23)	1.48 (±0.56)	36 (11)	-	0.23 (0.14–0.37)	1.95 (±0.49)	24 (11)	-
+PBO	260	0.02 (0.01–0.03)	1.70 (±0.23)	33 (23)	6	0.02 (0.01–0.04)	1.00 (±0.21)	50 (23)	11.5
+DEM	201	0.02 (0.01–0.06)	0.95 (±0.26)	47 (16)	6	0.03 (0.01–0.08)	0.85 (±0.22)	33 (16)	7.7

^1^ Synergistic Ratio (SR_50_) was calculated by dividing the KD_50_/LD_50_ obtained with no synergist divided by the KD_50_/LD_50_ obtained with the synergist.

**Table 5:** Toxicity and synergism of permethrin (PM) and etofenprox (EF) against *An. gambiae* (Strain A*kdr*).

	n	KD_50_, ng/mg (95% CI)	Slope (±SEM)	χ^2^ (df)	SR ^1^	RR ^2^	LD_50_, ng/mg (95% CI)	Slope (±SEM)	χ^2^ (df)	SR ^1^	RR ^2^
PM	405	1.67 (1.26–2.32)	2.36 (±0.16)	110 (34)	-	12.8	1.83 (1.33–2.70)	1.62 (±0.25)	81 (34)	-	14.1
+PBO	266	0.29 (0.14–1.40)	0.93 (±0.25)	50 (19)	5.8	5.8	0.37 (0.19–1.41)	0.84 (±0.20)	31 (19)	4.9	18.5
+DEM	193	0.45 (0.36–0.58)	2.15 (±0.26)	17 (16)	3.7	4.5	0.21 (0.15–0.28)	1.58 (±0.23)	21 (16)	8.7	3.0
EF	166	1.12 (0.68–2.83)	2.01 (±0.51)	43 (15)	-	9.3	0.99 (0.59–2.45)	1.86 (±0.46)	41 (15)	-	4.3
+PBO	270	0.62 (0.43–0.97)	1.63 (±0.25)	38 (20)	1.8	31	0.37 (0.22–0.65)	1.56 (±0.32)	64 (20)	2.7	18.5
+DEM	267	0.47 (0.33–0.68)	2.37 (±0.42)	59 (19)	2.4	23.5	0.76 (0.50–1.32)	1.53 (±0.31)	46 (19)	1.3	25.3

^1^ Synergistic Ratio (SR_50_) was calculated by dividing the KD_50_/LD_50_ obtained with no synergist divided by the KD_50_/LD_50_ obtained with the synergist; ^2^ Resistance Ratio (RR_50_) was calculated by dividing the KD_50_/LD_50_s obtained with the A*kdr* strain from the G3 strain.
